# Study on Green Degradation Process of Polyurethane Foam Based on Integral Utilization and Performance of Recycled Polyurethane Oil-Absorbing Foam

**DOI:** 10.3390/ma15124269

**Published:** 2022-06-16

**Authors:** Shu Peng, Depeng Gong, Youliang Zhou, Chaocan Zhang, Yinchun Li, Chunyang Zhang, Yitian Sheng

**Affiliations:** School of Materials, Science and Engineering, Wuhan University of Technology, Wuhan 430070, China; ps15771189488@163.com (S.P.); gdpyy1@163.com (D.G.); zyl_2013@whut.edu.cn (Y.Z.); liyinchun@whut.edu.cn (Y.L.); 13476089628@163.com (C.Z.); chrysemy@163.com (Y.S.)

**Keywords:** polyether polyol glycolysis, single-phase product, integral utilization, recycled foam, oil adsorption

## Abstract

Ester exchange glycolysis of flexible polyurethane foam (PU) usually results in split-phase products, and the recovered polyether polyols are obtained after separation and purification, which can easily cause secondary pollution and redundancy. In this paper, we propose a green recycling process for the degradation of waste polyurethane foam by triblock polyether, and the degradation product can be used directly as a whole. The polyurethane foam can be completely degraded at a minimum mass ratio of 1.5:1. The secondary full utilization of the degradation product as a whole was directly synthesized into recycled polyurethane foam, and the compression cycle test proved that the excess glycolysis agent had less effect on the resilience of the recycled foam. The hydrophobic modification of the recycled foam was carried out, and the oil absorption performance of the recycled foam before and after the hydrophobic modification was compared. The oil absorption capacity for diesel oil ranged from 4.3 to 6.7, while the oil absorption performance of the hydrophobic modified recycled foam was significantly improved and had excellent reusability (absorption–desorption oil processes can be repeated at least 25 times). This economical and green process has large-scale application prospects, and the hydrophobic recycling foam can be applied to the field of oil and water separation.

## 1. Introduction

Polyurethane, one of the most widely used polymers in the world [1,2,3], can be mainly divided into foams and CASEs (Coatings, Adhesives, Sealants, Elastomers). With such a large amount of polyurethane being produced and applied, it also brings the problem that a large amount of used polyurethane waste cannot be recycled and used, and nearly 10% of polyurethane waste can only be burned or landfilled, which has a huge impact on the environment [4]. With the growing concern of humans and the government about environmental issues, there is a lot of attention on how to recycle and utilize polyurethane waste properly.

The recycling of polyurethane is mainly divided into physical recycling and chemical recycling. The physical recycling method is to recycle polyurethane waste by rebonding, compression molding, regrinding or powdering polyurethane waste into pieces or powder as filler. These methods are simple and easy to implement and do not change the chemical structure of polyurethane, but the recycled polyurethane is only used in low-end products with poor product performance and its tendency to produce secondary waste [5]. Therefore, the chemical recovery method is a more potential recovery method. The chemical recovery method is divided into hydrolysis, aminolysis, phosphorolysis and glycolysis [6]. Among them, ester exchange glycolysis is the most important chemical recovery method. In the literature that has been reported, researchers are committed to using small molecular polyols to degrade polyurethane foams. The components of aromatic isocyanate derivatives generated by transesterification have poor solubility with recovered polyol components, so the split-phase products are obtained [7]. The upper layer is mainly composed of recovered polyols and a small amount of excess glycolysis agents. The lower layer contains most glycolysis agent polyols and the aromatic isocyanate derivatives generated by transesterification [8,9]. The main purpose of this series of studies is to recover polyether polyols in the upper product [9,10,11], so researchers attach great importance to the phase separation ability of the glycolysis agent [12]. The lower the content of the glycolysis agent in the upper layer is, the easier the subsequent separation and purification process. De Lucas et al. [13] investigated the degradability of various polyols and their ability to phase separate using diethanol (DEA) amine as catalyst and monoethylene glycol (MEG), diethylene glycol (DEG), 1,2-propanediol (MPG) and propylene glycol (DPG) as low weight glycols. Among them, DEG showed a good degradation capacity of the urethane chain, requires short time, and causes the best phase separation; in addition, the obtained product is most similar to the polyol of synthetic polyurethane. Molero et al. [14] further determined that the most suitable catalyst for DEG was stannous octanoate, and the highest quality polyol was obtained when stannous octanoate was used. Simon et al. [9] determined the optimum reaction conditions for DEG with stannous octanoate as degradation agent and catalyst, with a catalyst concentration of 1.3 wt %, a reaction temperature of 190 °C, and a mass ratio of DEG to polyurethane foam between 1:1.125 and 1.5. Although the split-phase products obtained by glycolysis using low weight glycols can recover high purity polyols and can be recycled, the split-phase glycolysis results in a large molar excess of glycols, making the actual cost of this method huge. Therefore, De Lucas et al. [15] used crude glycerol, which is inexpensive and easily available, as a degradant, thus greatly reducing the economic cost of split-phase glycolysis, and because of the large dielectric constant of crude glycerol, the by-product and degradant content of the upper phase was greatly reduced, and the lower layer was free of polyol, while the recovered high-quality polyol proved to be very useful for secondary use in the production of polyurethane foam. Del Amo et al. [7] expanded the split-phase glycolysis process using crude glycerol as a glycolysis agent from a laboratory scale to a pilot plant and obtained good economic benefits. However, in this method, the glycerol in the lower phase is difficult to separate from other recoverable substances because of its high boiling point, so the lower phase is prone to secondary contamination and waste. In the case of using glycol as glycolysis solvents, the glycolysis solvents with strong polarity are more capable of causing phase separation, and the glycolysis solvents have less residue in the upper layer [16], but they have less degradation ability compared to the lower polarity of glycol, while the less polar diol glycolysis solvents such as DEG are less capable of causing phase separation than the more polarity glycolysis solvents, but they have more degradation ability, take less time to degrade, and have more residues in upper layer. In a recent study, Thomas Veanbergen et al. [17] used 2-pyrrolidone as an additive that can effectively accelerate the dissolution and degradation rate of the glycolysis reaction. When the mass ratio of 2-pyrrolidone to PU foam is 0.1:1, the mass ratio of diol to PU foam can be reduced from 1.5:1 to 0.5:1 without affecting the purity and yield of the recovered polyether polyol. In the study of split-phase glycolysis with the main purpose of recovering polyether polyol from the upper material, how to obtain the upper phase with higher purity and lower glycolysis agent content, as well as to avoid the excessive cost caused by the huge molar excess of glycolysis agent is the main development direction of this study.

In this paper, a waste polyurethane degradation process with single-phase degradation products that can be directly used as a whole was proposed. The product does not need to be separated to avoid secondary pollution and reduce the cost. The flexible triblock polyether (PEG-PPG-PEG) as a glycolysis agent can be directly used to synthesize regenerated flexible polyurethane foams, which is expected to have little effect on the resilience of regenerated foams. Therefore, triblock polyether (PEG-PPG-PEG) with high active primary hydroxyl at both ends was used as a degradation agent for the transesterification glycolysis of flexible polyurethane foam. It is expected that the triblock polyether will be bonded to the aromatic isocyanate components by transesterification reaction, so as to increase the solubility of aromatic isocyanate components and polyether polyol components, and prepare single-phase degradation products that can be directly used as a whole to reduce secondary pollution. The glycolysis products were used as a whole, and the recycled polyurethane foam was synthesized by polyaryl polymethylene isocyanate (PAPI) reactions with glycolysis products. The foam has good resilience, and the surface hydrophobic modification of the recycled polyurethane foam was carried out. A reusable oil absorption material was obtained, which can be used for sewage treatment.

## 2. Materials and Methods

### 2.1. Materials

Haian Petroleum and Chemical Co. (Haian, China) supplied the triblock polyether (Triblock Polyoxypropylene Polyoxyethylene Copolymer, PEG-PPG-PEG) L31 (Mn = 1100 g/mol, functionality with respect to OH groups: 2) and polyether polyols (PPG) HSH-210 (Mn = 1000 g/mol, functionality with respect to OH groups: 2) as glycolysis agents, Dow Corning (Midland, MI, USA) supplied the amino silane 8040A for recycled foam surface modification, Wanhua Chemcial Group Co. (Yantai, China) supplied PAPI to synthesized polyurethane foam, Zibo Dihua PU Co. (Shandong, China) provided foam stabilizer DH3380, Yexing Co. (Ningbo, China) provided porosity-opening agent 3350 and Sinopharm Chemical Reagent Co. (Shanghai, China) provided the other raw ingredients.

### 2.2. Methods

#### 2.2.1. Glycolysis

The glycolysis reaction was carried out in a 500 mL three-neck flask. Firstly, the glycolysis agent was added to the three-neck flask and heated up to the reaction temperature of 180 °C by oil bath heating under the atmospheric pressure. Then, the waste PU was added to the three-neck flask, and the whole reaction was carried out under nitrogen atmosphere in order to avoid the side reaction of foam oxidation. The ratios of L31 to foam were 1:1, 1.5:1, and 2:1, and the reaction times were 4 h, 6 h, 8 h, 10 h, and 12 h, respectively. The ratios of HSH-210 to foam were 3:1 and 4:1, and the reaction times were 4 h, 6 h, 8 h, 10 h, and 12 h, respectively. The experiment of L31 was marked as in Table 1 with an HSH-210 to foam mass ratio of 4:1, reaction temperature of 180 °C, and reaction time of 12 h; the product is marked as D_1_.

#### 2.2.2. Synthetic Recycled PU Foam

Firstly, the hydroxyl value of the degradation product was determined by standard titration methods (ASTMD-4274-88), and the hydroxyl value of the product was 106.18 mgKOH/g. The PAPI dosage was calculated based on the hydroxyl value of the product.

(1) $$m\left(PAPI\right)=\frac{E\times m\left(\mathrm{product}\right)\times I\times X\left(OH\right)}{1000\times 56.11}$$

*E* is the equivalent value, and isocyanate index (*I*) is the molar ratio of NCO/OH of isocyanate to polyol from the -NCO content of PAPI, which is 30.5%; thus, we can calculate the equivalent value *E* = 42/30% = 137.7. Based on the above test, we can calculate the amount of PAPI to be added from the amount of degradation product and isocyanate index. The amount of PAPI to be added to the reaction can be calculated from the amount of degradation products and isocyanate index required by ourselves. Considering that the foam produced needs good elasticity and strength to meet the requirements of repeated use, the isocyanate index used in this experiment is 1.3, in which the excess isocyanate group can provide reaction sites for amino silane oil in the surface modification.

Before preparing polyurethane foam, all kinds of glycolysis products were firstly dehydrated to prevent the influence of water in the foaming process. This experiment adopts a one-step method to synthesize polyurethane foam. Firstly, mix degradation products, foam stabilizer (DH3380), porosity-opening agent (3350) and catalyst (DBTDL) according to the formula ratio and stir for 30 min; then, add foaming agent (141-b) and continue to stir for 20 min, until the material is mixed evenly. Add PAPI and stir rapidly for 10 s. The viscosity of the material increases sharply, and the reactions such as chain growth and cross-linking are completed in a short time. Then, heat the material in a water bath at 75 °C. Foaming is completed within 0.5–2.5 min to obtain polyurethane foam with a certain cross-linking degree, and then, the foam is put into a 90 °C oven to mature for 24 h.

#### 2.2.3. Amino Silane Modified Recycled PU Foam

The surface modification of polyurethane foam was carried out by using amino silane, and the amino group of amino silane reacted with the excess isocyanate group in polyurethane foam to graft on the surface of polyurethane foam to achieve the effect of hydrophobic modification. The reaction solution was firstly prepared by weighing the amino silicone oil with the same mass as the foam and then configuring the amino silane xylene solution with a volume ratio of 1:10. The reaction solution was heated up to 120 °C and added to the polyurethane foam for 2 h. After the reaction is completed, the polyurethane foam is washed with anhydrous ethanol and then dried in an oven at 60 °C.

### 2.3. Characterization

The viscosity of the degradation products was characterized using a rotational viscometer (model NDJ-1, Nanjing, China), the molecular weight and composition of the products were analyzed using Gel Permeation Chromatograph (GPC, model APC, Waters, Milford, MA, USA), and a cold field emission scanning electron microscope with an additional X-Max N80 energy spectrometer (SEM model JSM-7500F, Akishima, Japan) was used to characterize the micromorphology of the PU foam. The cyclic compression properties of the samples were determined using an electronic universal material testing machine (model Instron 5967, Norwood, MA, USA). A contact angle analyzer is used to measure the sample water contact angle (WCA model JC2000C, Shanghai, China). The hydroxyl number were determined by standard titration methods (ASTMD-4274-88).

### 2.4. Oil-Absorbing Capacity

First, 30 mL of oil was added to the beaker; gasoline, motor oil, and canola oil were used in this study. Recycled oil-absorbing PU foam was squeezed using tweezers and then placed in the oil to release it, left to make it absorb oil for 1 min, and then taken out to drain the oil for 30 s. The mass of the foam before oil absorption was recorded as *M*_1_, and the mass after oil absorption was recorded as *M*_2_. The ratio of oil absorption capacity *K* can be calculated from the following equation.

(2) $$K=\frac{M_{2}-M_{1}}{M_{1}}$$

All types of oils were repeatedly measured 5 times, and the average value was taken to obtain the final oil absorption capacity ratio.

### 2.5. Reusable Performance

In this study, the reusable properties of the material were tested by repeated absorption–desorption oil processes. High viscosity canola oil was chosen as the experimental oil for this experiment. The mass of the unabsorbed polyurethane foam material was first measured and then recorded with a balance. A sufficient amount of canola oil was placed in a beaker, and the prepared sponge was gently placed on the surface of the oil, absorbed for one minute and then removed with tweezers. After being drained for 30 s, the mass of the adsorbed saturated foam was measured with a balance and recorded. After the recording was completed, the oil in the polyurethane foam was squeezed out and the polyurethane foam was cleaned with petroleum ether and anhydrous ethanol and dried in a blast drying oven at 60 °C. The contact angle was tested after the drying was completed. The contact angle and oil absorption capacity of the foam were measured at different numbers of cycles of this process to determine the reusability of the recycled PU oil-absorbing foam.

## 3. Results and Discussion

### 3.1. Degradation and Characterization of Polyurethane Foams

#### 3.1.1. Degradation Performance of Tirblock Polyether (PEG-PPG-PEG) and Polyether Polyols (PPG)

**Figure 1:**
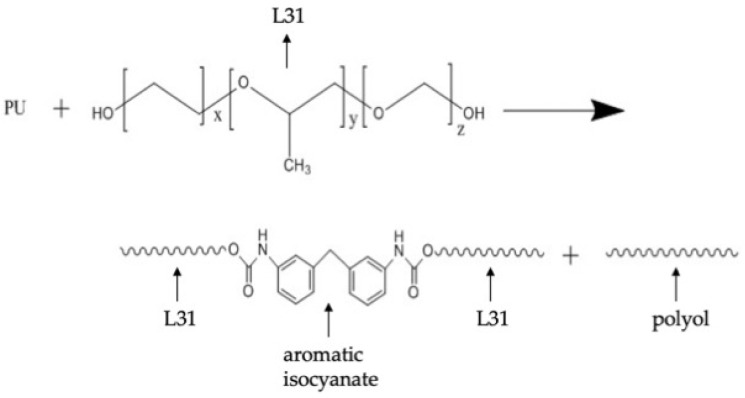
The reaction mechanism of transesterification reaction when L31 is glycolysis agent.

In order to achieve the overall use of polyurethane foam degradation products, polyether polyol was used as a glycolysis agent. It was expected that the triblock polyether was bound to aromatic isocyanate components through ester exchange reaction so as to improve the solubility of aromatic isocyanate components in the free polyol (the reaction mechanism is shown in Figure 1). Generally, the reactivity of the polymer is lower than that of small molecules, so the triblock polyether (PEG-PPG-PEG, functionality with respect to OH groups: 2, L31) containing high reactive primary hydroxyl groups at both ends is used to improve the reaction efficiency, and the degradation effect of polyether polyols HSH-210 (one end is primary hydroxyl and the other is secondary, hydroxy functionality with respect to OH groups: 2) and triblock polyether on polyurethane foam was compared.

Table 2 describes the degradation products of L31 and HSH-210.

It can be seen from Table 2 that single-phase products were obtained by using L31 and HSH-210 to glycolysis PU flexible foam, which is completely different from the reported split-phase glycolysis [18]. When L31 is used as a glycolysis agent, it is easier to obtain single-phase products with longer reaction time at the same mass ratio, because the longer reaction time makes the degradation of polyurethane foam more complete. When the mass ratio of L31 to polyurethane foam was 1:1, only 12 h of reaction was available to obtain the single-phase products, and when the reaction time is 2 h, PU cannot be completely degraded, as the product contains solid material. While 8 h and more of reaction time could obtain the single-phase products when the mass ratio was 1:1.5, when the reaction time is 2 h, the product contains solid material. After 6 h and more of reaction time, the single-phase products could be obtained when the mass ratio was 2:1, indicating that a larger mass ratio with a larger amount of glycolysis agent could provide an accelerated reaction, and an excess of glycolysis agent can act as a solvent in the system, allowing better solubilization of the aromatic isocyanate polymer modified by L31. When HSH-210 was used as a glycolysis agent, when the mass ratio was less than 3:1, HSH-210 could not effectively degrade the polyurethane foam even after 12 h of reaction. When the mass ratio was 3:1, the homogeneous product could not be obtained, and the products with reaction time less than 8 h contained solid substances. When the mass ratio was 4:1, the products with reaction time less than 6 h contained solid substances, and the single-phase product could be obtained after 12 h of reaction. Compared with L31, when the reaction time is the same, HSH-210 needs four times the weight of L31 as a glycolysis agent to obtain single-phase products, indicating that the reaction activity of L31 is much higher than that of HSH-210.

The viscosity of the L31 glycolysis product was further tested for the reaction time of 4 h and above, and the viscosity of the product under the same conditions was measured to reflect the extent of decomposition of the polyurethane foam and the reaction rate in that reaction time period. After the reaction was completed, the product was transferred into a beaker, cooled to 25 °C (±1 °C), and then the viscosity of the product was tested, and the average value was taken after three tests.

**Figure 2:**
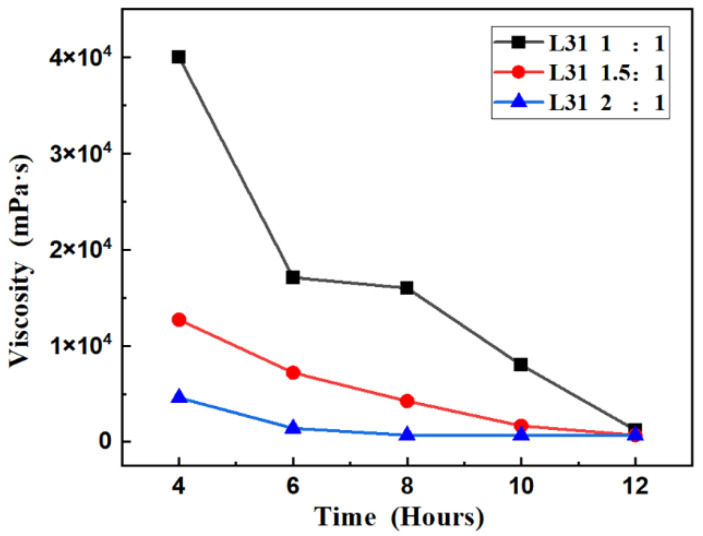
Viscosity of glycolysis product.

The viscosity test of the three groups of experiments A, B and C is shown in Figure 2. In the experiment of group A, the viscosity of the product was 40,000 mPa·s when the mass ratio of L31 to foam was 1:1, the reaction temperature was 180 °C, and the reaction time was 4 h, which indicated that the reaction was not high when the reaction was only 4 h, and there were still more long chains that had not been reacted, and the molecular weight of the product was larger. After continuing the reaction, the viscosity of the product decreased sharply, and the viscosity of the product was 17,100 mPa·s after 6 h of reaction; however, the viscosity of the product decreased to 16,000 mPa·s after 8 h of reaction, indicating that the reaction speed slowed down in this time period. The viscosity of the product was 8000 mPa·s and 1250 mPa·s when the reaction time is 10 h and 12 h, respectively. It can be seen from the change of viscosity that the reaction was the fastest between 4 and 6 h and the viscosity decreased most obviously, and in this system, the viscosity of the system was still changing when reaction time is 12 h, so it cannot indicate that the reaction was carried out to the end point.

The mass ratio of L31 to foam in group B experiments was 1.5:1 and the reaction temperature was 180 °C. The viscosity of the product was 12,700 mPa·s at the reaction time of 4 h. Compared with A1, the viscosity of the system was lower, which was not only because the larger mass ratio provided more L31 as a solubilizer to reduce the viscosity of the whole system but also because the larger mass ratio led to the increase in the molar ratio of block polyether to foam in the system, and the larger reaction speed was obtained. The viscosity of the product was 4350 mPa·s at 8 h, and the reaction speed was still fast. The viscosity of the product was 1675 mPa·s at 10 h and 700 mPa·s at 12 h, and the reaction speed decreased from 8 to 12 h. The viscosity of the system was still changing when the reaction time was 12 h, so it cannot indicate that the reaction was carried out to the end point.

In group C experiments, the mass ratio of L31 to foam was 2:1, and the reaction temperature was 180 °C. The viscosity of the product was 4600 mPa·s when the reaction time was 4 h, and the viscosity of the product continued to decrease after 2 h: from 4600 to 1425 maP.s. The reaction speed during the period from 4 to 6 h compared with the subsequent reaction speed was the fastest, while the reaction 8 h after the viscosity gradually stabilized; the 8 h, 10 h, and 12 h product viscosity values were 700 mPa·s, 712.5 mPa·s, and 705 mPa·s, respectively. At 8 h to 12 h, the reaction speed slowed down; after the 8 h reaction ended, the molecular weight of the system tended to stabilize, and there was no major change.

#### 3.1.2. Molecular Weight and Composition Analysis of Degradation Products

GPC was used to analyze the molecular weight and composition of each group of products as well as the L31 and foam mass ratio of 1:1 reaction for 12 h products, 1:1.5 reaction for 12 h products, 2:1 reaction for 8 h and 12 h products, namely A_5_, B_5_, C_3_, and C_5_. For an HSH-210 to foam mass ratio of 4:1, reaction temperature of 180 °C, and reaction time of 12 h, the product is marked as D_1_, and GPC tests were performed on these products.

**Figure 3:**
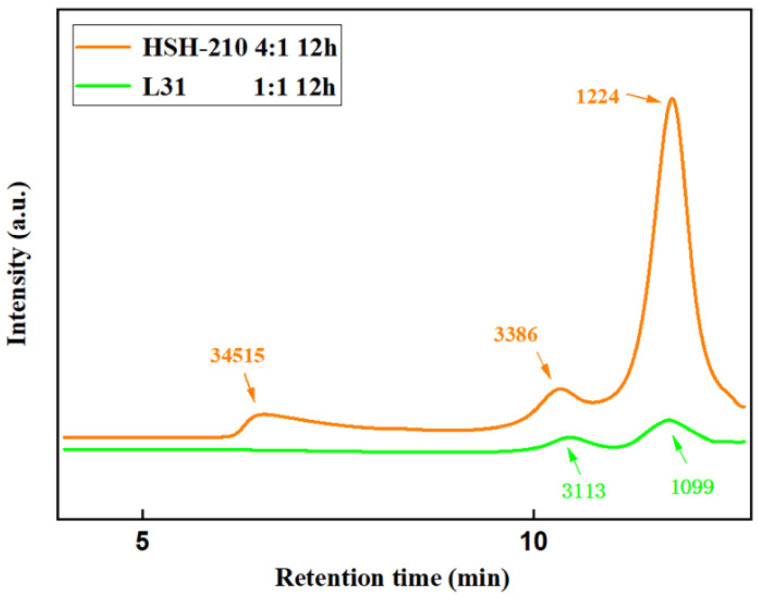
GPC chromatograms of D_1_ product and A_5_ product.

The comparison graph of GPC between the product of group D_1_ and the product of A_5_ is shown in Figure 3. Comparing the GPC images of the two products, a peak with a molecular weight of 34,515 appears in the product of D_1_ compared with the product of A_5_, which is divided into incompletely degraded polyurethane foams containing multiple structural units of polyurethane, so the molecular weight is larger, indicating that even though HSH-210 used such a large mass ratio, there was a large molar excess in the system and at the reaction time of 12 h, HSH-210 still could not completely degrade the foam, and there were still some parts that were not degraded. In comparison with the product of group A_5_, the mass ratio of block polyether to foam was only 1:1 at the same reaction temperature and reaction time, and the molar excess was much smaller than that of group D_1_, but it was still able to degrade the polyurethane foam completely. This phenomenon indicates that the reactivity of L31 with the secondary hydroxyl group at both ends is much higher than that of the HSH-210 with the primary hydroxyl group at both ends, so L31 is chosen to degrade the polyurethane foam with a larger reaction rate, and a better degradation effect can be obtained without a large molar excess. In the product, the peak of molecular weight of about 3100 is composed of recycled polyols with a molecular weight of about 3100 and an aromatic isocyanate polymer modified by L31 and HSH-210, while the peak of molecular weight of about 1100 is composed of excess glycolysis agent and recycled polyether polyols with a molecular weight of about 1000 in polyurethane foam.

**Figure 4:**
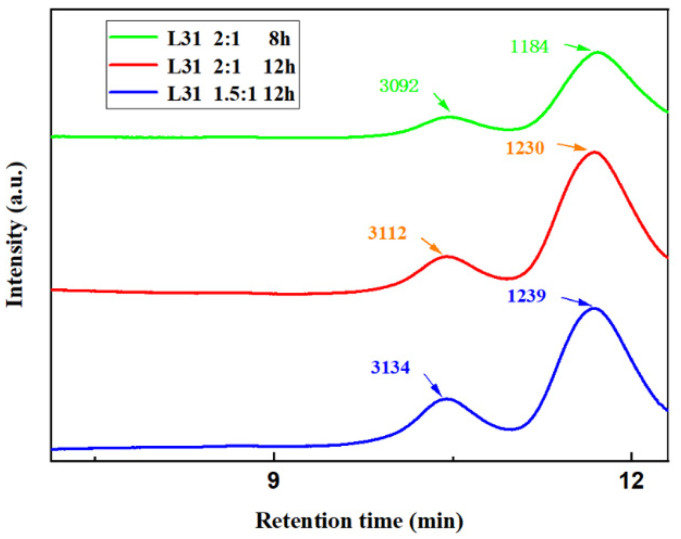
GPC chromatograms of C_3_, C_5_ and B_5_ product.

The GPC values of the products of C_3_, C_5_ and B_5_ experiments are compared in Figure 4, through which we can see that both C_3_ and C_5_ experiments completely degraded the polyurethane foam. Comparing the GPCs of these two products, it can be found that there is no significant difference in the molecular weight and content of the products of the C_3_ and C_5_ groups at the extended reaction time, which indicates that the reaction has reached the end point at 8 h, and there is no significant change in the products when the reaction time is extended, which reflects the previous analysis of the viscosity of the products of the C group. The GPC images of the experimental products of group B_5_, which did not show any product with larger molecular weight, indicated that the polyurethane foam was completely degraded and the reaction reached the end point, which was similar to the values of each component in the other three products, indicating that the L31 effectively degraded the polyurethane foam under the reaction conditions.

### 3.2. Study on Recycled PU Foam and Its Oil Absorption Performance

In recent years, along with the continuous and rapid economic development, human beings have produced a large amount of oil-containing sewage in production and life, and the problem of environmental pollution has become increasingly prominent, so the development of oil-absorbing materials to deal with pollution containing cannot be delayed [19,20,21,22].

The hydroxyl value of B_5_ groups of experimental degradation products was determined to be 106.18 mgKOH/g by hydroxyl value titration. The amount of PAPI was calculated according to the hydroxyl value, and the recycled flexible polyurethane foam was prepared by PAPI curing. Then, the surface of the recycled foam was hydrophobically modified by amino silane to obtain the hydrophobic porous oil-absorbing recycled polyurethane foam. The microstructure, oil absorption performance and reuse performance were characterized.

#### 3.2.1. Micromorphology of Recycled PU Foam

**Figure 5:**
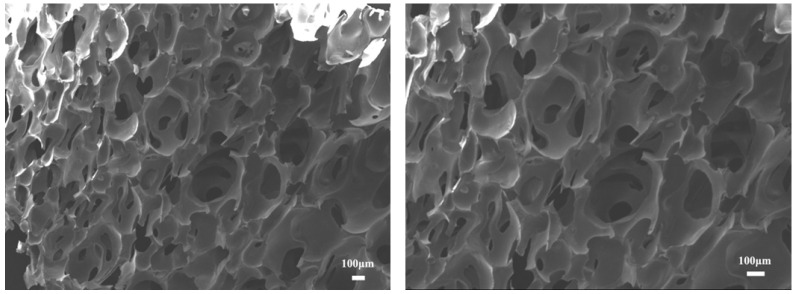
Scanning electron microscopy (SEM) images of recycled PU foam.

Firstly, the microstructure of recycled foam was observed to determine whether its pore structure can be applied to the oil absorption field. The SEM images of recycled polyurethane foam are shown in Figure 5.

As can be seen from the figure, the recycled polyurethane foam has an obvious honeycomb structure, which contains a large number of pores, and these pores have considerable advantages in the field of oil absorption, which can help play a role in storing oil when it is applied to oil–water separation. Moreover, the pores of polyurethane foam are divided into open pores and closed pores. Closed pore polyurethane foam has an independent pore structure, and the pores are not connected with each other and cannot pass through gas or liquid, while open pore polyurethane foam is connected with each other and can pass through gas or liquid, so open pore polyurethane foam can effectively adsorb and store oil when it is used in the field of oil–water separation. We can observe from this SEM image that this polyurethane foam has an obvious open pore structure, and the pores are connected to each other and can pass through liquids, so it has a greater potential to adsorb and store oil when applied in the field of oil–water separation.

#### 3.2.2. Hydrophobic Performance of Recycled PU Oil-Absorbing Foam

**Figure 6:**
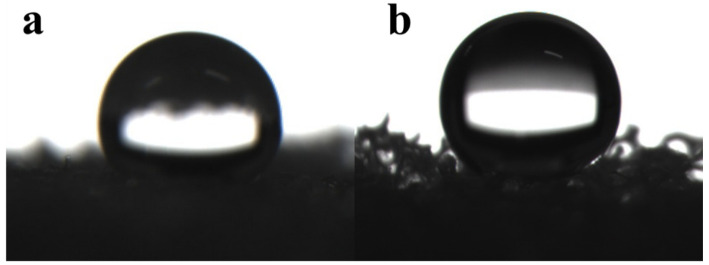
Water contact angle (WCA) of recycled PU (**a**) WCA of recycled PU oil-absorbing foam (**b**).

Hydrophobic property is an important indicator of whether the material can be applied to the field of oil absorption; therefore, the contact angle meter was used to measure the water contact angle more for the polyurethane foam before and after modification. As shown in Figure 6, the water contact angle of the pre-modified polyurethane foam is about 120°, which is greater than 90°, indicating that the foam has a certain hydrophobic performance, but its hydrophobic performance is not strong enough to be applied to oil–water separation. In contrast, the water contact angle of the modified hydrophobic polyurethane foam can reach 147°, indicating that the grafting of amino silicone oil on the surface of the polyurethane foam gives it more excellent hydrophobic properties, and it also indicates that the foam can be applied to oil–water separation.

#### 3.2.3. Compression Performance of Recycled Oil-Absorbing PU Foam

**Figure 7:**
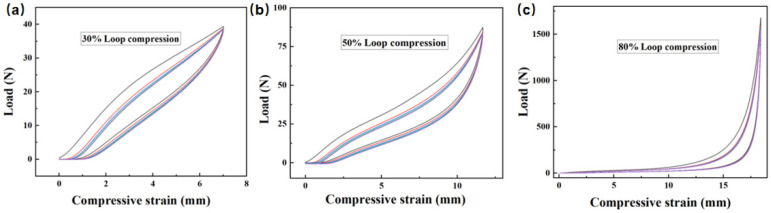
(**a**) Stress–strain curves for cyclic compressive 30% deformation of modified PU foam. (**b**) Stress–strain curves for cyclic compressive 50% modified PU foam. (**c**) Stress–strain curves for cyclic compressive 80% modified PU foam.

Polyurethane foam has great potential as a reusable oil-absorbing material because of its excellent elasticity and mechanical properties. Therefore, the modified polyurethane foam was tested for a stress–strain cycle curve of compression deformation under different strains and to investigate whether it can be used as a reusable oil-absorbing material. The modified polyurethane foam was tested for five cycles of compressive deformation at 30%, 50%, and 80% strains, respectively, and this result is shown in Figure 7. The modified polyurethane foam still has good elasticity and compressibility, which lays the foundation for its use as an oil-absorbing recycled polyurethane foam. In the 10 compression cycles at 30%, 50%, and 80% strain, there is a certain loss of elasticity of the modified polyurethane foam, but all of them reach stability after the 3rd loop, which indicates that it meets the basic performance requirements as a reusable oil-absorbing material and can repeat absorption–desorption oil processes by compression and release. At the same time, it is further explained that using triblock polyether as a glycolysis agent, even if the L31 has a certain degree of excess, when the degradation products are directly used as a whole, excessive L31 does not affect the resilience of the recycled foam.

#### 3.2.4. Oil-Absorbing Capacity of Recycled Oil-Absorbing PU Foam

The oil absorption capacity of a material is an important technical indicator to evaluate its application in the field of oil–water separation. In this study, gasoline, engine oil, and canola oil were used, and the recycled oil-absorbing polyurethane foam was squeezed with tweezers, placed in the oil and released, and left to absorb oil for 1 min; then, it was taken out and drained for 30 s and weighed, and the ratio of oil absorption capacity K obtained based on mass was calculated. The adsorption capacity ratios K for gasoline, engine oil and canola oil are shown in the Table 3. It can be seen that the pre-modified foam can adsorb 4.7 times its mass of gasoline, while the modified hydrophobic foam can adsorb 6.2 times its mass of gasoline. For diesel oil, the pre-modified foam can adsorb 4.3 times its mass of engine oil, while the modified foam can adsorb 6.7 times its mass of engine oil. For canola oil, the foam before modification can adsorb 3.1 times its mass of canola oil, and after modification, it can adsorb 4.8 times its mass of canola oil. The oil-absorption rate of the recycled oil absorption foam is relatively low compared with the results reported in the literature, which is caused by the lower porosity of the recycled foam caused by the preparation process of the recycled foam [23]. However, it can be clearly seen from these data that the polyurethane foam modified by amino silicone oil has a large improvement in its oil absorption capacity and its ability to store oil, and the recycled polyurethane oil-absorbing foam prepared by the modification has good oil absorption capacity, which is sufficient for application in the field of oil–water separation.

#### 3.2.5. Reusable Performance

In addition to its large pore volume for oil storage, polyurethane foam also has excellent elasticity and compressibility, which allows the modified polyurethane foam to undergo repeated absorption–desorption oil processes by repeated squeezing. This is a great advantage of polyurethane foam for oil–water separation applications, so it is also important to test its reusability. In this study, the reusability performance of the material was tested by repeated absorption–desorption oil processes.

The values of the oil absorption capacity ratio K and water contact angle of this material after 5, 10, 15, 20 and 25 times of absorption–desorption oil processes, respectively, are shown in Table 4.

As we can see from the table, the contact angle of the recycled polyurethane oil-absorbing foam decreased slightly during the process of repeated absorption–desorption oil from 5 times to 25 times, but its oil adsorption capacity did not change much in general, indicating that the repeated oil absorption and oil removal process did not damage the adsorption capacity of the modified polyurethane foam, which has the performance of repeated use.

#### 3.2.6. Oil–Water Separation Selection Performance

**Figure 8:**
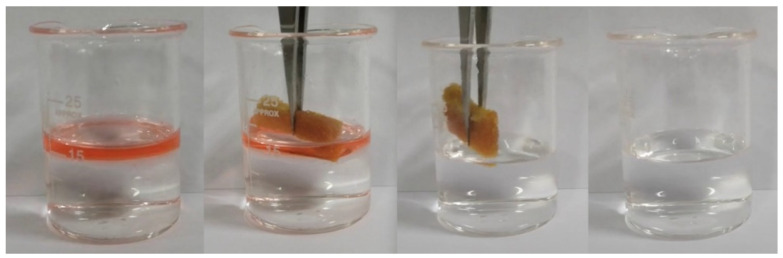
Optical images for absorption oil process of the oil-absorbing PU foam.

The process of oil absorption is shown in Figure 8. It can be seen that the oil-absorbing polyurethane foam immediately absorbs the oil when it touches the oil, and due to the oleophilic and hydrophobic property of this oil-absorbing polyurethane foam, water cannot enter the pores of this foam, and the oil can be quickly absorbed by this foam. Then, the oil-absorbing polyurethane foam moves in the water and absorbs the oil completely to obtain a clean water surface to achieve the effect of oil–water separation.

**Figure 9:**
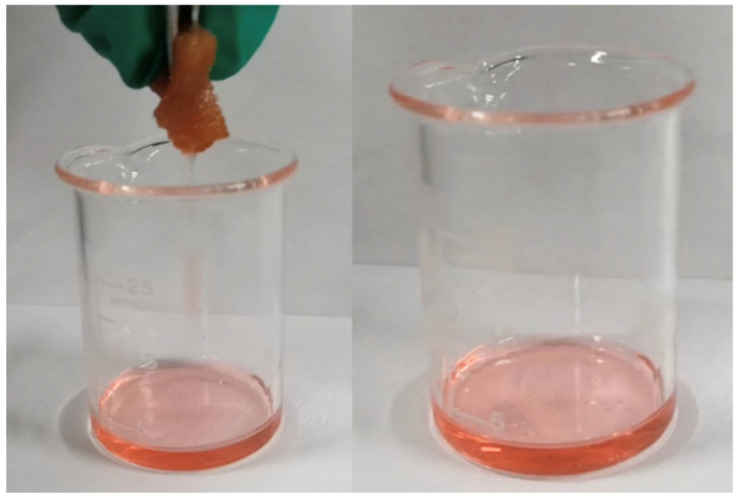
Optical images for desorption oil process of the oil-absorbing PU foam.

Figure 9 shows the desorption oil process of the oil-absorbing PU foam. By squeezing the foam, the recovery of oil and the reuse of the oil-absorbing polyurethane foam can be achieved simply and easily, and from the recovered oil, it can be found by visual observation that there is no water in the recovered oil, indicating that the oil-absorbing PU foam has good selectivity for separating the oil–water mixture.

## 4. Conclusions

The complete degradation of polyurethane foams was effectively carried out using triblock polyether, and homogeneous products were obtained that could be directly utilized as a whole. The optimum reaction conditions for the degradation of polyurethane foam by triblock polyethers were investigated as follows: dibutyltin dilaurate as catalyst, 1% mass ratio, 1.5:1 mass ratio of triblock polyether to polyurethane foam, 180 °C, and 12 h reaction time. Through the ester exchange reaction, the triblock polyether effectively modified the aromatic isocyanate component, increased the molecular weight of the aromatic isocyanate component, increased its compatibility with the polyether polyol component, and prepared a homogeneous degradation product that can be directly used as a whole to reduce secondary pollution.

The recycled polyurethane foam was synthesized by the secondary integral direct utilization of degradation products and cured with PAPI, and the recycled oil-absorbing polyurethane foam was prepared by hydrophobic modification of the recycled foam with amino silane. The water contact angle is 147°, and it can adsorb 6.2 times its weight of gasoline, 6.7 times its weight of motor oil, and 4.8 times its weight of canola oil, which are all greatly improved compared with those before modification. It can repeat absorption–desorption oil processes 25 times, and it has excellent selectivity for oil–water mixtures. This green process in which degradation products can be integrally utilized will effectively reduce the cost of polyurethane degradation and recycling, and the hydrophobic recycling foam can be applied to the field of oil and water separation.

## Figures and Tables

**Table 1:** The serial number of L31 experiment.

Glycolysis Agent		L31		
	Mass Ratio	1:1	1.5:1	2:1
Time				
4 h		A_1_	B_1_	C_1_
6 h		A_2_	B_2_	C_2_
8 h		A_3_	B_3_	C_3_
10 h		A_4_	B_4_	C_4_
12 h		A_5_	B_5_	C_5_

**Table 2:** Phenomenon of degradation products of L31 and HSH-210.

Glycolysis Agent		L31			HSH-210	
	Mass Ratios	1:1	1.5:1	2:1	3:1	4:1
Time						
2 h		Split *	Split *	Split	Split *	Split *
4 h		Split	Split	Split	Split *	Split *
6 h		Split	Split	Single-phase	Split *	Split *
8 h		Split	Single-phase	Single-phase	Split *	Split
10 h		Split	Single-phase	Single-phase	Split	Split
12 h		Single-phase	Single-phase	Single-phase	Split	Single-phase

* Product contained solids matter.

**Table 3:** Ratio of oil absorption capacity *K* for different oil products before and after modification.

Oils	Gasoline	Diesel Oil	Canola Oil
*K*_1_ (before modification)	4.7	4.3	3.1
*K*_2_ (after modification)	6.2	6.7	4.8

**Table 4:** Ratio of oil absorption capacity K and water contact angle (WCA) of polyurethane foam after repeated absorption–desorption oil processes.

Times	5	10	15	20	25
K	4.9	5.28	5.21	4.8	4.7
WCA	143.6°	145.7°	146.4°	145.5°	144.6°

## Data Availability

The data presented in this study are available on request from the corresponding author.
